# The structure and configuration changes of multifunctional peptide vectors enhance gene delivery efficiency†

**DOI:** 10.1039/c8ra04101f

**Published:** 2018-08-07

**Authors:** Sen Yang, Zhao Meng, Ziyao Kang, Chao Sun, Taoran Wang, Siliang Feng, Qingbin Meng, Keliang Liu

**Affiliations:** State Key Laboratory of Toxicology and Medical Countermeasures, Beijing Institute of Pharmacology and Toxicology Beijing 100850 China nankaimqb@sina.com keliangliu55@126.com +86-10-68211656 +86-10-68169363

## Abstract

We designed a series of peptide vectors that contain functional fragments with the goal of enhancing cellular internalization and gene transfection efficiency. The functional fragments included a cell-penetrating peptide (R_9_), a cationic amphiphilic α-helical peptide [(LLKK)_3_-H_6_ or (LLHH)_3_], a stearyl moiety, and cysteine residues. Vectors were also synthesized with D-type amino acids to improve their proteolytic stability. The conformations, particle sizes, and zeta potentials for complexes of these peptides with pGL3 plasmid DNA were characterized by circular dichroism and dynamic light scattering. In addition, cellular uptake of the peptide/DNA complexes and gene transfection efficiency were investigated with fluorescence-activated cell sorting and confocal laser-scanning microscopy. Greater transfection efficiency was achieved with the vectors containing the R_9_ segment, and the efficiency was greater than Lipo2000. In addition, the D-type C_18_-c(llkk)_3_ch_6_-r_9_ had about 7 times and 5.5 times the transfection efficiency of Lipo2000 in 293T cells and NIH-3T3 cells at the N/P ratio of 6, respectively. Overall, the multifunctional peptide gene vectors containing the R_9_ segment exhibited enhanced cellular internalization, a high gene transfection efficiency, and low cytotoxicity.

## Abstract 2

The R_9_ containing peptide vectors can improve the gene transfection efficiency.

## Graphical abstract

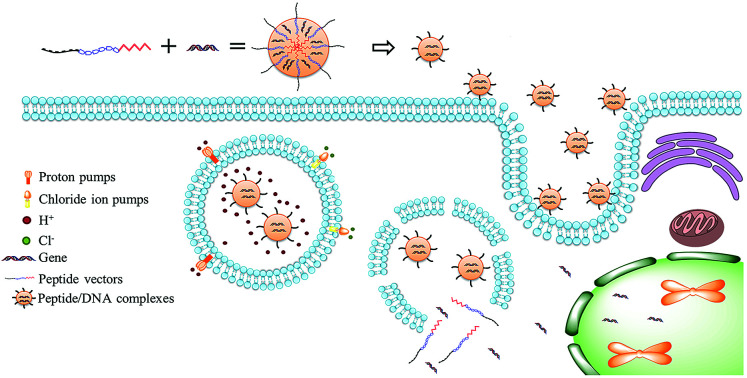

## Introduction

Gene therapy has been investigated for its potential to treat genetic diseases such as cancer, severe combined immunodeficiency, cystic fibrosis, and various monogenic diseases. Based on advances made in this field, the therapeutic potential of gene therapy in the clinic has gained significant attention over the past three decades.^1,2^ The effective components of gene therapy include oligonucleotides, DNA and RNA, which can exhibit high specificity and low toxicity in mediating their therapeutic effects.^3^ Unfortunately, poor permeability of the plasma membrane to gene molecules, as well as the sensitivity of oligonucleotides to enzymatic degradation *in vivo*, has substantially complicated the development of oligonucleotide molecules for therapeutic applications.^4^ Thus, it is necessary to build delivery vector systems which ensure that gene molecules are able to enter cells and be effective.

Generally, there are two types of gene delivery vectors: viral and non-viral. The former are most frequently used in clinical therapies based on their excellent delivery efficiency. However, viral vectors have also exhibited high cytotoxicity and limited immunogenicity in clinical applications.^5^ Jesse Gelsinger underwent viral vector gene therapy, was the first reported death due to this type of treatment.^6^ The subsequent increase in concern regarding patient safety led to increased consideration of non-viral vectors, including cationic liposomes,^7^ polymers,^8^ nanoparticles,^9^ peptides,^10^ and multifunctional envelope-type nano devices (MEND),^11^ for the delivery of gene molecules. Ideally, a gene vector should be safe and efficient while overcoming barriers and obstacles such as DNA combination, cellular uptake, endosomal escape, and cytoplasmic mobility before gene molecules reach the nucleus.^12^

Among the different kinds of vectors that have been developed, peptide vectors have gained widespread popularity as a very promising non-viral vector in recent years. Moreover, various functional segments have been incorporated into peptide vectors to affect the cell penetration,^13^ targeting,^14^ and endosomal escape^15^ properties of these vectors. For example, cell penetrating peptides (CPPs) consisting of positive lysine and arginine residues can impart cationic properties to a peptide vector to facilitate interactions with lipid plasma membranes.^16^ A minimal basic peptide domain sequence from the trans-activator of transcription (TAT) of HIV, GRKKRRQRRR, has been shown to bind many types of cargo *via* non-covalent or covalent interactions and undergo translocation across plasma membranes *via* the endocytosis passway.^17,18^ Wender *et al.* employed a systematic reverse engineering approach to demonstrate that the ability of TAT to enter cells is not a function of its peptide backbone, but rather is a function of the number and spatial array of its guanidinium groups.^19^ Similarly, limited sequences of arginine (R_6_–R_12_) or lysine (K_6_–K_12_) residues have exhibited cell penetrating properties, with the penetrating efficiency of poly-arginine peptides being greater than that of poly-lysine peptides.^20–22^ Rothbard *et al.* further demonstrated that R_9_ was taken up by cells more quickly than TAT.^23^ However, while TAT and poly-arginine (R_6_–R_12_) peptides are able to penetrate the cell membrane, they have not exhibited good gene transfection efficiency. Similarly, while CPPs are taken up by cells, their uptake is often non-selective. As a result, the number of CPPs in circulation is reduced and only a small percentage of CPPs accumulate in their target cells.^24^ CPP-mediated endocytosis has also been shown to result in the localization of complexes to endosomes, which leads to their eventual degradation in lysosomes.^25^ Consequently, a combination of functional segments appears to be necessary for achieving effective gene delivery.

To date, the challenges that are associated with gene delivery remain varied and complicated, although a key challenge is endosomal escape.^26^ Vector/DNA complexes are internalized *via* trafficking through endosomes (endocytosis) and are eventually degraded in lysosomes.^27^ During this process, the pH of the trafficking endosomes continuously decreases.^28^ To facilitate the escape of vector/DNA complexes from endosomes, a ‘proton sponge effect’ has been considered whereby agents with a high buffering capacity and an ability to swell when protonated are used. Histidine-rich materials have also exhibited an ability to escape from endosomes due to the imidazole ring they contain.^29–31^ Previously, (LLHH)_3_ and (LLKK)_3_-H_6_ have been used as functional segments in peptide gene vectors to enhance the endosomal escape and transfection efficiency of these vectors. These segments exhibited an ability to disrupt endo-lysosomal membranes and mediate a ‘proton sponge effect’.^32,33^

Hydrophobic modifications of cationic peptide vectors can also enhance the cellular uptake and endosomal escape of complexes by facilitating interactions with membranes *via* hydrophobic moieties.^34,35^ For example, a stearyl moiety at the N-terminus of cationic peptide vectors has been shown to increase α-helicity and enhance gene delivery.^36^ In an aqueous environment, cationic amino acids of amphiphilic peptide vectors extend into the aqueous phase, while stearyl moieties will self-assemble inside vector structures.^37^ As a result, the vectors will form a secondary amphiphilic α-helical structure which enhances the binding ability of plasmid DNA and cellular uptake efficiency.^38^ The incorporation of cysteine residues into peptides can also increase α-helical content, potentially according to the sequence of the peptide molecule. Moreover, cysteine residues can increase the potential for intermolecular crosslinking to occur *via* disulfide bonds in acidic endosomes or lysosomes, thereby improving peptide/DNA complex stability in an extracellular environment. For example, when cysteine residues were used to link low molecular weight peptides *via* reducible disulfide bonds, cellular uptake and gene transfection efficiency were enhanced.^39,40^ It has also been reported that cysteine residues can affect the gene delivery performance of conjugates. For example, transfection of a cysteine-containing peptide was found to be approximately 7 times more efficient than a glycine-containing peptide that was used as a control.^41^

In this article, we describe the design and solid-state synthesis of a series of peptide vectors for gene delivery. The peptides include kinds of functional segments including: (I) a non-arginine segment for DNA binding, penetration of the cell membrane, and to facilitate peptide internalization; (II) an α-helical endo-lysosomal membrane disrupting (ELMD) segment for endosomal escape; (III) a stearyl moiety to promote self-assembly *via* a hydrophobic environment and improvements in α-helical conformation; and (IV) cysteine residues to improve peptide/DNA complex stability. Previously it was reported that D-type peptides are more stable than L-type peptides due to their reduced degradation by enzymes.^23,42^ Therefore, we hypothesized that the introduction of D-type amino acids would improve the stability of our peptide vectors both *in vitro* and *in vivo*, thereby enhancing delivery efficiency. The size, zeta potential, transfection efficiency, and cytotoxicity of our panel of peptide vectors in complex with a pGL3 were evaluated. The stabilities of these peptide vectors were also examined in the presence of proteinase K.

## Materials and methods

### 2.1. Materials and reagents

*N*-Fluorenyl-9-methoxycarbonyl (Fmoc)-protected L-type amino acids and D-type amino acids were purchased from GL Biochem Ltd. and CS Bio Ltd. (Shanghai, China), respectively. Rink amide resin (0.44 mmol g^−1^) was purchased from Nankai Hecheng (Tianjin, China). 2-(1*H*-Benzotriazole-1-yl)-1,1,3,3-tetramethyluroniumhexauorophosphate (HBTU) and hydroxybenzotriazole (HOBT) were purchased from GL Biochem Ltd. Diisopropylethylamine (DIEA), trifluoroacetic acid (TFA), thioanisole, ethandithiol, and anisole were purchased from J & K Scientific (Beijing, China). All other solvents were redistilled from drying reagents.

Dulbecco's Modified Eagle Medium (DMEM) and fetal bovine serum (FBS) were purchased from Gibco (CA, USA). Penicillin–streptomycin, phosphate-buffered saline (PBS), YOYO-1 (491/501) intercalating dye, and Lipofectamine 2000 (Lipo2000) were purchased from Invitrogen (CA, USA). A pGL3 control vector, Dual-Glo® Luciferase Assay System, and CellTiter 96® Aqueous One Solution Cell Proliferation Assay were purchased from Promega (WI, USA). A Bradford protein assay kit was purchased from Beijing Solarbio Science & Technology Co. Ltd. (Beijing, China).

### 2.2. Synthesis and purification of peptides

All peptides were synthesized by using solid-phase synthesis with Fmoc chemistry on an automatic Liberty-12-Channel Automated Peptide Synthesizer with an integrated microwave system (CEM, North Carolina, USA). Rink amide resin (0.57 g) was suspended in 5 mL anhydrous *N*,*N*-dimethylformamide (DMF) in a centrifuge tube (50 mL) which was connected to the automated peptide synthesizer. The appropriate amino acids and HBTU/HOBT/DIEA were added to the centrifuge tube automatically, and a 20% solution of piperdine in DMF (v/v) was applied to the resin to remove the Fmoc protected groups. Amino acids were coupled to the rink amide resin one by one, followed by N-terminal conjugation of a stearic acid. Finally, a cleavage reagent (10 mL) containing TFA (90%)/thioanisole (5%)/ethandithiol (3%)/anisole (2%) (v/v/v/v) was freshly prepared for deprotection of the side chains and cleavage of the target peptides from the resin over 0.5 h at 0 °C and 2.5 h at room temperature. Crude peptides were then purified with reverse-phase high-performance liquid chromatography (HPLC). A gradient of acetonitrile and deionized (DI) water containing 0.1% (v/v) TFA was applied to a C8 or C18 column (Waters, Massachusetts, USA). Molecular weights (MWs) of the generated peptides were analyzed by electrospray ionization mass spectrometry (ESI-MS) or matrix-assisted laser desorption/ionization time-of-flight mass spectrometry (MALDI-TOF-MS).

### 2.3. Circular dichroism (CD) measurements

The secondary structures of the synthesized peptides were determined from CD spectra. Briefly, peptide solutions (50 µM) were prepared in 50% trifluoroethanol/PBS (v/v) solution to imitate the cell membrane environment. CD spectra were recorded with a Biologic MOS-450 instrument (Claix, France) between 190 nm and 280 nm at room temperature. Experimental conditions included: a speed of 50 nm min^−1^, a 2 s time response, 0.5 nm resolution, 4.0 nm bandwidth, and a 1.0 mm path cell. All spectra were converted to a uniform scale after background subtraction. The reported curves were smoothed with standard parameters.

### 2.4. Preparation of vector/pGL3 complexes

Peptide/DNA complexes were prepared at various charge ratios (N/P) by mixing 0.9 µg pGL3 control DNA with the corresponding amount of peptide solution in 50 µL PBS. Mixtures were vortexed for 10 s, incubated at 37 °C for 30 min to form complexes, and then were diluted to 900 µL with serum-free DMEM culture medium. As a positive control, 1 µg of pGL3 control DNA (in 50 µL PBS) was mixed with 2.5 µL Lipo2000 (1.0 mg mL^−1^). For a negative control, 0.9 µg unmodified pGL3 DNA was added to 900 µL of serum-free DMEM.

### 2.5. Agarose gel electrophoresis assay

Peptide/DNA complexes at N/P ratios ranging from 0 to 3.5 were prepared with 0.5 µg pGL3 plasmid DNA in solution. Complexes were then diluted to 10 µL with PBS, and 2 µL DNA loading buffer was added to provide a DNA dye. After the complexes were loaded on a 1% (w/v) agarose gel with tris-acetate-ethylenediamine tetra-acetic acid buffer, they were separated at 100 V for 45 min. The gels were stained with 300 mL ethidium bromide solution (0.5 µg mL^−1^) for 30 min and imaged with a ChampGel 6000 system (Beijing Sage Creation Science and Technology Co., Ltd., Beijing, China).

### 2.6. Size and zeta (ζ) potential measurements of the peptide/DNA complexes

Peptide/DNA complexes were prepared at N/P ratios of 2 to 8 with 100 µL of DNA solution (containing 2.0 µg pGL3) in the corresponding amount of peptide solution with the same volume. After 30 min at 37 °C, dynamic light scattering (DLS) (Zetasizer Nano ZS90, Malvern, UK) was performed at 25 °C with a scattering angle of 90° to measure the size of the peptide/DNA complexes. The complex mixtures were then diluted to 1.0 mL with deionized water to measure the ζ-potential of each. Each experiment was repeated three times.

### 2.7. Transmission electron microscopy (TEM)

To investigate the morphologies of the peptide/DNA complexes, the complexes at an N/P ratio of 6 were observed with TEM (Hitachi H-7650, Tokyo, Japan). Briefly, the samples were prepared as described above, with 1.0 µg pGL3 added into the appropriate peptide solution. The mixtures were diluted to 500 µL with deionized water, incubated for 30 min at room temperature, and then added dropwise onto a copper TEM grid that could hold approximately 10 µL of each sample per well. After the samples air dried they were examined.

### 2.8. Cell culture and cytotoxicity assay

293T cells and NIH-3T3 cells were cultured in DMEM containing 10% FBS and 1% penicillin–streptomycin (1 × 10^4^ U mL^−1^) at 37 °C with 5% CO_2_.

Cytotoxicity of the peptide/DNA complexes was evaluated in both 293T cells and NIH-3T3 cells with a CCK-8 kit (Dojindo). Briefly, cells were initially seeded into a 96-well plate (Greiner Bio One, Germany) at a density of 1.0 × 10^5^ cells per mL with 100 µL cells added per well. After being cultured for 24 h, 100 µL FBS-free medium was added to each well and peptide/DNA complexes containing 0.2 µg of DNA at N/P ratios ranging from 2 to 8 were added. After 4 h, the medium was removed and 100 µL DMEM containing 10% FBS was added to each well. After an additional incubation at 37 °C in a 5% CO_2_ atmosphere for 20 h, 10 µL CCK-8 reagent was added to each well. After 2 h at 37 °C, absorbance values were measured at 450 nm with a SpectraMax® M5 instrument (Molecular Devices, WI, USA). Cells without complexes were included as a negative control. Lipo2000/DNA complexes (prepared as described above) were included as a positive control. Experiments were repeated five times for each sample.

### 2.9. *In vitro* transfection

To evaluate the transfection capacity of the peptide vectors *in vitro*, a reporter gene method was used. Briefly, 300 µL of 293T cells and NIH-3T3 cells at a density of 3.0 × 10^4^ cells in DMEM containing 10% FBS were added into individual wells. After 24 h, the medium was removed and peptide/DNA complexes were added to each well in fresh DMEM. After an additional 4 h, the medium was replaced with DMEM containing 10% FBS and cells were incubated for 44 h before being assayed. The Lipo2000/DNA complex was used as a positive control.

For the luciferase assays performed, the medium in each well was removed and the cells were washed 3× with PBS. A cell culture lysis reagent (50 µL per well; Promega) was then added to each well. After 15 min at room temperature, the plates were centrifuged at 4 °C (4000 rpm) for 8 min and 20 µL of each cleared supernatant was collected and transferred into the wells of a new plate. Reagent (100 µL) from a luciferase assay kit (Promega) was added to each well. Relative light units were recorded by a SpectraMax® M5 instrument set for a 500 ms read time. This same sample preparation method was used to perform the endocytosis inhibition studies, except that the cells were pretreated with chloropromazine hydrochloride (CPZ, 10 µg mL^−1^), amiloride (50 µM), or methyl-beta-cyclodextrin (MβCD, 5 mM).

### 2.10. Fluorescence-activated cell sorter (FACS) analysis

293T cells were seeded into 6-well plates with DMEM containing 10% FBS at a density of 2.0 × 10^5^ cells per well and were incubated for 24 h at 37 °C with 5% CO_2_. For labeling, pGL3 (2.0 µg) was incubated with 5 µL YOYO-1 for 30 min at 37 °C. The peptides plus labeled pGL3 complexes were then added to each well. After 4 h, the medium was removed and each well was washed 3 times with PBS. The cells were treated with 200 µL of a 0.25% (w/v) trypsin/0.02% (w/v) EDTA solution until approximately 80% of the cells in each well detached from the plate. Then, 800 µL of DMEM containing 10% FBS was added to each well and the samples were collected into individual 1.5 mL centrifuge tubes. After a 5 min centrifugation step at 900 rpm, each supernatant was removed and resuspended in 200 µL PBS. A minimum of 15 000 events per sample were analyzed by a FACS instrument (Becton Dickinson, NJ, USA) at an excitation/emission ratio of 488 nm/530 nm.

### 2.11. Confocal laser-scanning microscopy (CLSM)

293T cells were seeded into 15 mm glass-bottom culture dishes containing DMEM/10% FBS at a density of 3.0 × 10^4^ cells per well and incubated at 37 °C with 5% CO_2_. After 24 h, YOYO-1-labeled DNA/peptide complexes were freshly prepared as described above, and then added to each well after its medium was removed. After 4 h, each well was washed 3 times with PBS and then fixed with PBS containing 4% formaldehyde. After 10 min, the wells were washed 2 times with PBS before cell nuclei were stained with DAPI (2.0 µg mL^−1^, Roche, Switzerland). After the cells underwent two more washes with PBS, they were examined under a Carl Zeiss LSM 510 meta fluorescence microscope (Jena, Germany) with long focal length optics and excitation by He–Ne (543 nm) and argon ion (488 nm) lasers.

For live-cell imaging experiments, YOYO-1-labeled pGL3 plasmid DNA was incubated with corresponding peptides for 30 min. Then, Lyso-Tracker Red (50 nM) was incubated with the cells for 30 min to stain endosomes and lysosomes. After the cells were washed with PBS, they were incubated with Hoechst 33258 (1 µg mL^−1^) for 30 min to stain cell nuclei. After a final wash with PBS, the cells were examined for approximately 2 h with an Ultra VIEW VoX live-cell imaging system (Perkin Elmer, US) equipped with a modular laser system that uses solid-state laser technology.

### 2.12. Enzymatic hydrolysis stability

Peptides and proteinase K were dissolved in PBS and then diluted to 8.0 mg mL^−1^ and 2.0 mg mL^−1^, respectively. For each peptide tested, 0.5 mL of peptide solution was mixed with 0.5 mL proteinase K solution and then incubated in a water bath at 37 °C. After 0, 5, 15, 30, 60, and 120 min, a 10 µL sample of the mixture was withdrawn and 30 µL of a precipitator solution (1.0 mL of TFA in 9.0 mL acetonitrile) was added. Each sample was subsequently vortexed for 5 s and centrifuged for 10 min at 4 °C. Supernatants were analyzed by HPLC, with the peak area of each peptide recorded and used to calculate hydrolysis efficiency.

### 2.13. Statistical analysis

Data were recorded as the mean standard deviation (SD) of triplicate groups (*n* = 3). Differences in data were statistically determined by using one way analysis of variance (ANOVA). A *p*-value ≤ 0.05 indicated a difference, a *p*-value ≤ 0.01 indicated a significant difference, and a *p*-value ≤ 0.001 indicated a highly significant difference.

## Results and discussion

### 3.1. Design, synthesis, and characterization of a panel of peptide vectors

The peptide vectors generated for this study contained four functional segments: a CPP segment (R_9_ or TAT), an ELMD segment [(LLHH)_3_ or (LLKK)_3_-H_6_], a stearyl segment, and cysteine residues. The sequences of these peptides are listed in Table 1. For the peptides containing the CPP segment, R_9_, these were named P-01 to P-04. The peptides, P-05 and P-06, contained the other CPP segment, TAT, and have been previously described.^32^ P-02, P-04, and P-06 were synthesized with D-type amino acids, while the remaining peptides included L-type amino acids. All of the peptides were synthesized by using a solid-phase synthesis method with Fmoc chemistry. The peptides were subsequently purified and analyzed by HPLC (purity ≥ 95%) (Fig. S1†). Mass spectrometry confirmed the MWs of the peptides (Fig. S2†).

**Table 1:** Sequences and molecular weights of the peptides designed and synthesized in this study^a^

Compounds	Peptide sequences	Molecular weight	
		Calculated	Measured
P-01	C_18_-CLLHHLLHHLLHHC-RRRRRRRRR	3397.2	3396.1
P-02	C_18_-cllhhllhhllhhc-rrrrrrrrr	3397.2	3396.1
P-03	C_18_-CLLKKLLKKLLKKC-HHHHHH-RRRRRRRRR	4166.3	4164.7
P-04	C_18_-cllkkllkkllkkc-hhhhhh-rrrrrrrrr	4166.3	4164.7
P-05	C_18_-CLLHHLLHHLLHHC-GRKKRRQRRR	3370.2	3371.7
P-06	C_18_-cllhhllhhllhhc-grkkrrqrrr	3370.2	3369.0

a All of the peptides were purified and analyzed by HPLC (purity ≥ 95%). MWs of peptides, were confirmed by MS (data are shown in Fig. S1 and S2).

### 3.2. CD measurements

**Fig. 1:**
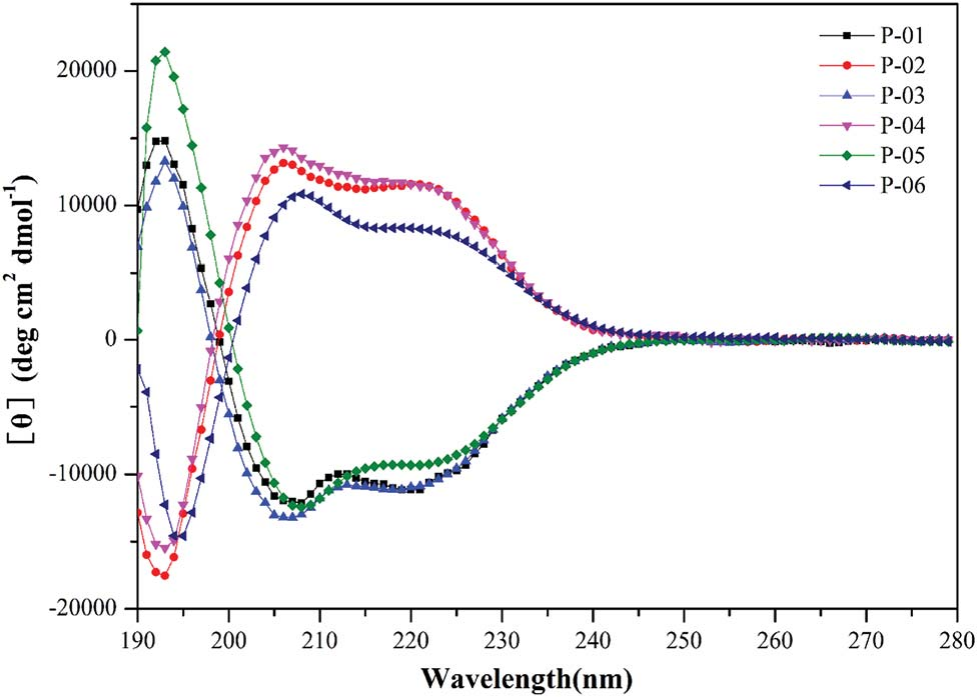
CD spectra of peptide vectors (P-01 to P-06) at 50 µmol L^−1^ in 50% trifluoroethanol/PBS.

It has been demonstrated that peptides that form an α-helix can disrupt endosomal membranes and improve the endosomal escape efficacy of peptide/DNA complexes.^43^ To determine whether the R_9_ segment affects the ability of a peptide to form an α-helical structure, CD spectra were obtained for P-01, P-02, P-03, and P-04 in a 50% trifluoroethanol/PBS (v/v) solution that was used to simulate a membrane environment. The peptides, P-05 and P-06 (which contain the CPP segment, TAT) were included as controls. The spectra of all six peptides exhibited a minimal absorbance peak at 208 nm and a pronounced shoulder at 220 nm (Fig. 1), both of which are typical characteristics of an α-helical conformation. Thus, all six peptide vectors were predicted to be able to disrupt endosomal membranes. The peptides containing a R_9_ segment also exhibited greater α-helicity than the TAT-containing peptides. The percentages of α-helicity^44^ for the peptides examined are listed in Table S1.† The conformation of the D-peptides was also confirmed to be opposite of the L-peptides, consistent with the different conformations of the amino acids used.^45^ Thus, the presence of a R_9_ segment in our peptide vectors induced an α-helical confirmation in the secondary structure of our chimeric peptide vectors.

### 3.3. Agarose gel electrophoresis assays

**Fig. 2:**
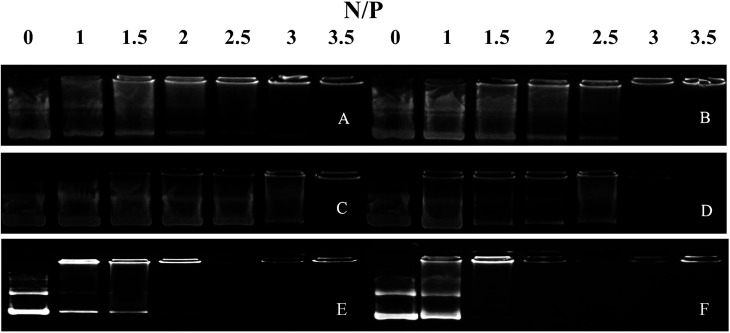
Agarose gel electrophoresis assays were conducted for all of the peptide vectors at various N/P ratios. (A) P-01, (B) P-02, (C) P-03, (D) P-04, (E) P-05, and (F) P-06.

We performed agarose gel assays to examine the ability of our peptides to complex with DNA. If the peptides were able to complex with a pGL3 vector at a reasonable N/P ratio, a band would be observed at the original loading site. In contrast, uncomplexed DNA would migrate toward the positive electrode. The D-peptides, P-02 and P-04, exhibited a greater capacity to bind DNA than the L-peptides, P-01 and P-03 (Fig. 2). Moreover, P-01 and P-03, and P-02 and P-04, exhibited similar DNA binding capacities among each of the two sets of peptides at N/P ratios of 3.5 and 3, respectively. The DNA-binding capacities of P-05 and P-06 were also similar at an N/P ratio of 2.5. In contrast, the peptide vectors containing the ELMD segments, (LLHH)_3_ and (LLKK)_3_-H_6_, did not exhibit any significant DNA binding capacity. Previously, Chuah *et al.* demonstrated that peptide vectors containing lysine and histidine residues could retard DNA migration at an N/P ratio of 5.^46^ Meanwhile, peptides that did not contain any lysine or histidine residues did not bind DNA even at a N/P ratio of 20.^46^

### 3.4. Particle size and zeta potential

**Fig. 3:**
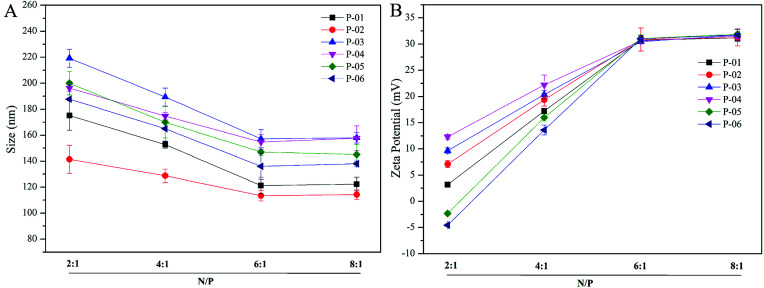
Particle size (A) and zeta potential (B) were measured for all of the peptide/DNA complexes indicated at different N/P ratios.

Particle size and zeta potential of peptide/DNA complexes play important roles in gene delivery by influencing cellular uptake, nuclear entry, and transfection efficiency.^43^ When our peptide vectors were prepared at N/P ratios ranging from 2 to 6, their sizes were found to decrease (Fig. 3). When they were prepared at N/P ratios ranging from 6 to 8, their sizes remained nearly unchanged. The sizes of the P-03/DNA and P-04/DNA complexes were larger than the other peptide/DNA complexes, and this might be due to their relatively high hydrophilicity that derives from the extra six lysines that are present in the ELMD segment they contain. Overall, our results indicate that our designed peptides were able to self-assemble into compact complexes with vector DNA at an N/P ratio of 6.

Positive charges that are present on the surfaces of peptide/DNA complexes affect endocytosis into the cytoplasm and gene transfection efficiency. For the peptide/DNA complexes examined in the present study, their positive charges increased when their N/P ratios were increased from 2 to 6, yet they remained unchanged when their N/P ratios were further increased from 6 to 8 (Fig. 3B). At an N/P ratio of 6, the peptides formed condensed DNA complexes that were smaller in size and tightly self-assembled to shield most of the positive charges of the complexes from excess peptides. This explanation is consistent with our particle size data (Fig. 3). Gong *et al.* previously reported that peptide vector/DNA complexes can form stable compounds when their N/P ratio is optimized.^47^ The size and zeta potential of their stable complexes were 80 nm and 27 mV, respectively.

### 3.5. Morphology

**Fig. 4:**
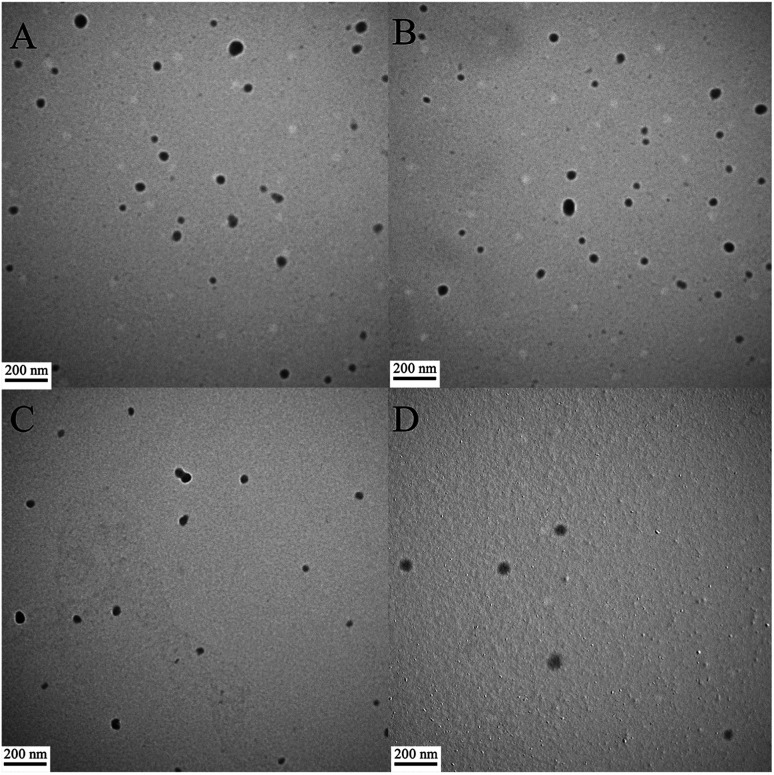
TEM images of peptide/DNA complexes prepared at an N/P ratio of 6. (A) P-01, (B) P-02, (C) P-03, and (D) P-04.

The morphologies of the peptide/DNA complexes prepared at an N/P ratio of 6 were observed by TEM (Fig. 4). Many of the complexes exhibited a spherical shape, and their sizes ranged from 40–80 nm. The average size of the complexes as measured by TEM was smaller than the size measured by DLS due to differences in testing conditions. For example, TEM was performed under dry conditions, whereas DLS was performed under liquid conditions. As a result, the DLS sizes refer to hydrodynamic diameters.^37^ All of the peptide/DNA complexes examined formed spherical nanoparticles *via* self-assembly, and the sizes of these peptides as measured by TEM did not show significant differences.

### 3.6. Transfection efficiency

**Fig. 5:**
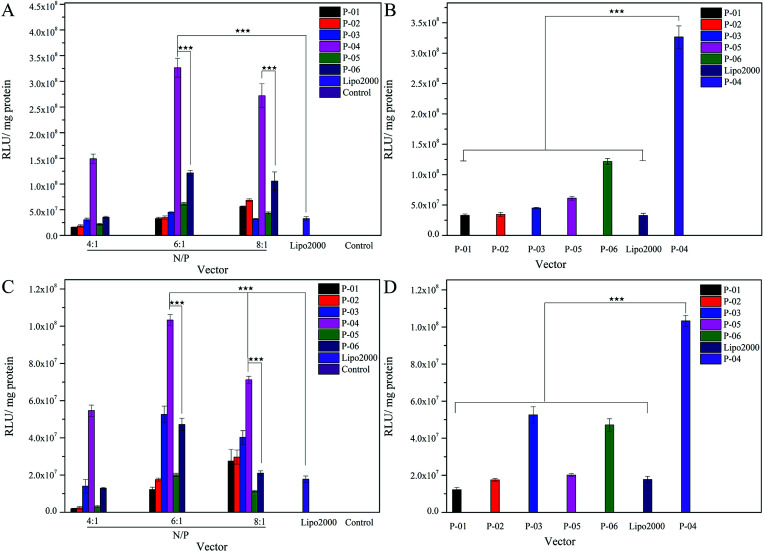
Expression levels of luciferase detected in 293T and NIH-3T3 cells treated with peptide/DNA complexes at various N/P ratios (A and C) and at an N/P ratio of 6 (B and D), respectively in each case. Data are presented as the mean ± SD (*n* = 3). DNA independent of peptide vector serves as a control. *p < 0.05; **p < 0.01; ***p < 0.005.

The delivery capacity of our designed peptide vectors at various N/P ratios were evaluated in 293T cells and NIH-3T3 cells in luciferase assays. Lipo2000 was included as a positive control. As shown in Fig. 5A and C, all of the vectors achieved successful gene transfection, and the transfection efficiency of most of the vectors was equal to or better than Lipo2000 at an N/P ratio of 6. Moreover, compared with the L-peptide vectors, the D-peptide vectors exhibited greater transfection efficiency at all of the N/P ratios examined. These results are consistent with previously reported observations that D-peptides are more stable than L-peptides and they are more resistant to intracellular degradation.^45^ The relatively high α-helicity content of the D-peptides may also enhance the endosomal escape of these peptides *via* disruption of endosome membranes. For P-01 and P-02, when their N/P ratio was increased from 4 to 8, their transfection efficiency increased. The transfection efficiency of peptides P-03 through P-06 also increased as their N/P ratio increased from 4 to 6. However, the transfection efficiency of these peptides slightly decreased when the N/P ratio ranged from 6 to 8. Overall, peptides P-03 through P-06 exhibited optimal gene transfection at an N/P ratio of 6. Correspondingly, the sizes and zeta potentials of these peptide/DNA complexes at this N/P ratio were found to be stable, as described above (Fig. 3). Peptide P-04 exhibited the greatest transfection efficiency, which was 7-fold greater than Lipo2000 and 2.5-fold greater than P-06 in 293T cells. Meanwhile, the transfection efficiency of peptide P-04 was 5.5-fold greater than Lipo2000 and 2.2-fold greater than P-06 in NIH-3T3 cells (Fig. 5B and D). Interestingly, the D-peptides, P-02 and P-04, which both contained an R_9_ segment, exhibited differences in their transfection abilities. The transfection efficiency of P-02 was equivalent to that of Lipo2000 at an N/P ratio of 6, and was higher than Lipo2000 at an N/P ratio of 8 in both cell lines. Structural differences between P-02 and P-04 derive from the presence of the endosomal escape segments, (LLHH)_3_ and (LLKK)_3_-H_6_, respectively. According to our previous work, when both the TAT segment and (LLHH)_3_ are present in a peptide, transfection is enhanced.^32^ Similarly, P-06 exhibited greater transfection efficiency compared with P-02 in the present study. Moreover, when the CPP segment was R_9_, the presence of (LLKK)_3_-H_6_ contributed to enhanced transfection efficiency. In contrast, naked DNA was not substantially delivered into either cell line (blank position in Fig. 5).

**Fig. 6:**
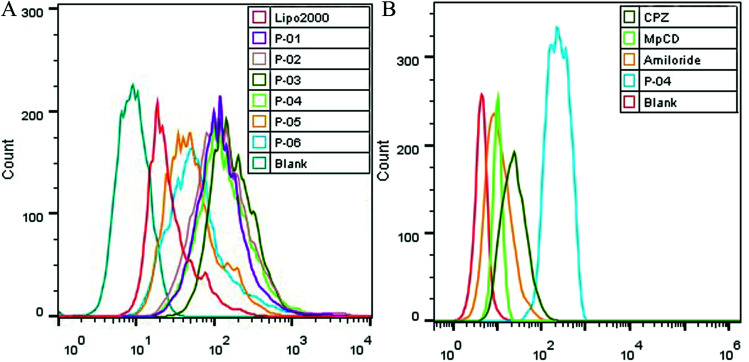
FACS assays were performed to detect cellular uptake. The uptake of various peptide/DNA complexes all at an N/P ratio of 6 were compared with Lipo2000 in 293T cells (A). In addition, uptake of P-04 at an N/P ratio of 6 with and without endocytosis-specific inhibitors pretreatment was observed (B). The pGL3 plasmid used was labeled with YOYO-1.

**Fig. 7:**
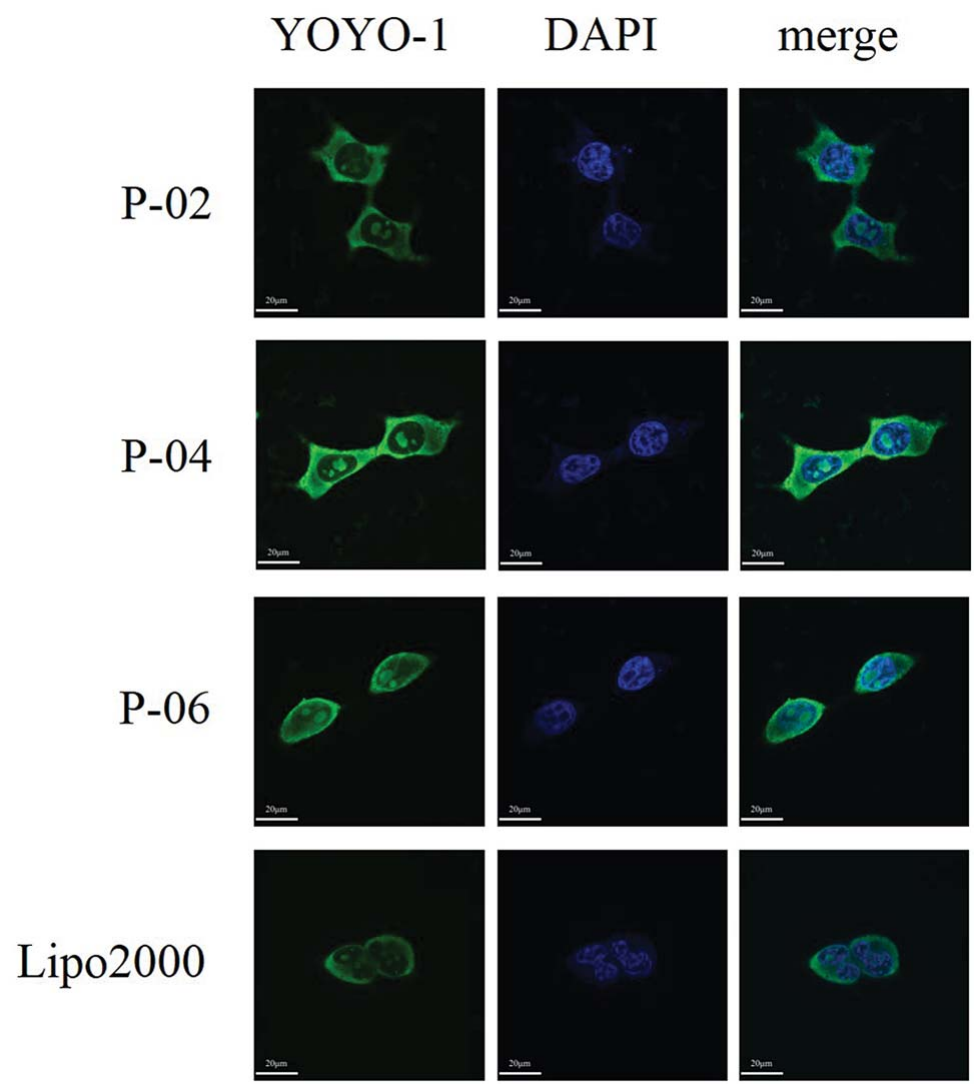
CLSM images of cellular uptake after peptide/DNA and Lipo2000/DNA complexes at an N/P ratio of 6 were applied to 293T cells for 4 h. The pGL3 plasmid was labeled with YOYO-1. Nuclei are stained with DAPI.

**Fig. 8:**
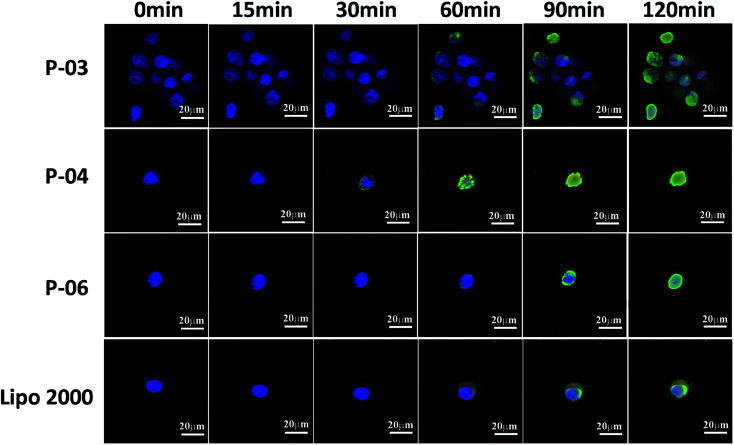
Cellular uptake and endosomal escape of P-03, P-04, and P-06/DNA complexes at a N/P ratio of 6 in 293T cells. Lipo2000/DNA complexes served as a positive control. CLSM images were obtained after incubating the peptide/DNA complexes with cells for 0, 15, 30, 60, 90, and 120 min intervals. The pGL3 plasmid was labeled with YOYO-1. Nuclei are stained with Hoechst 33258. Endosomes/lysosomes are stained with Lyso-Tracker Red.

It was observed that transfection efficiency did not markedly vary, nor did it decrease, as the N/P ratio increased from 6 to 8. These phenomena are likely due to the limited contribution of excess peptide to gene delivery and transfection in the context of DNA-binding saturation. Overall, P-04 achieved the highest transfection efficiency, with each functional segment of P-04 contributing individually and in a synergistic manner as well. At the cellular level, transfection efficiency increased as a result of cellular uptake, α-helical content, and endosomal escape. As shown in Fig. 1, the α-helical content of P-04 was similar to that of the other peptides. Therefore, in this case, transfection efficiency appears to depend on cellular uptake and endosomal escape. It was previously demonstrated that the efficiency of cell entry for peptides containing R_9_ was better than that of peptides containing TAT.^23^ When we examined cellular uptake efficiency with CLSM and in a live-cell imaging system, P-04 exhibited the best cellular uptake efficiency compared with the other peptides (Fig. 7 and 8). These results are consistent with those of previous reports where arginine–histidine–cysteine peptide systems were shown to be more efficient *in vitro*.^48^

### 3.7. Cellular uptake and translocation of peptide/DNA complexes

Cellular uptake is a key factor in gene delivery, thereby largely affecting transfection efficiency. To investigate the cellular uptake efficiencies of peptide/pGL3 complexes, both FACS and CLSM were performed. In the FACS experiments, all of the peptide vectors delivered YOYO-1-labeled pGL3 plasmid DNA into 293T cells at an optimal N/P ratio of 6. P-04 exhibited a high efficiency of cell entry compared to the other vectors and Lipo2000 (Fig. 6A). In addition, the R_9_-containing peptide vectors, P-01 to P-04, showed higher cellular uptake efficiency than P-05 and P-06. The latter results may be due to the capacity for the R_9_ residues to facilitate cell entry compared with TAT. Overall, peptides P-01 through P-06 all exhibited better delivery efficiency than Lipo2000.

Next, possible cellular uptake mechanisms were investigated with use of various endocytosis specific inhibitors: CPZ (to inhibit clathrin-mediated endocytosis), MβCD (to inhibit caveolin-mediated endocytosis), and amiloride (to inhibit caveolin-mediated macropinocytosis). Briefly, P-04 was selected to deliver YOYO-1-labeled pGL3 plasmid DNA into 293T cells at an optimal N/P ratio of 6. All three inhibitors listed above were found to significantly decrease the cellular uptake efficiency of the P-04/DNA complexes (Fig. 6B). These results indicate that peptide/DNA complexes can be internalized *via* various pathways, although the P-04/DNA complexes were mainly taken up by cells *via* endocytosis and macropinocytosis, possibly due to the size of the complexes. It has previously been reported that nanoparticles approximately 120 nm in diameter are internalized into cells *via* caveolin-mediated endocytosis, while particles with diameters of 50–70 nm are internalized *via* clathrin-mediated endocytosis.^49,50^ Furthermore, larger particles are internalized *via* macropinocytosis.^51^ The size of P-04 as determined with DLS was approximately 120 nm at a N/P ratio of 6. However, aggregation of self-assembled complexes can also occur, thereby leading to the formation of larger particles. Therefore, it is hypothesized that the main routes of cell entry for the P-04/DNA complexes are endocytosis and macropinocytosis.

To visualize peptide delivery efficacy, CLSM studies were performed. Briefly, 293T cells were incubated with YOYO-1-labeled peptide/pGL3 complexes for approximately 4 h. After staining the nuclei with DAPI, the best cell uptake efficiency was exhibited by P-04, followed by P-06, P-02, and Lipo2000. These results are consistent with the FACS data shown in Fig. 6A.

To investigate the rate of cellular uptake and endosomal escape, live-cell imaging experiments were performed. The nuclei and lysosome/endosome components of 293T cells were stained with Hoechst 33258 and Lyso-Tracker Red, respectively, for 30 min. YOYO-1-labeled DNA/peptide complexes were subsequently incubated with the cells before the cells were observed with CLSM. Signal appeared around the lysosomes and in the endosomes within 30 min of staining the P-04-treated cells (Fig. 8). These results indicate that P-04 delivered the labeled plasmid DNA into the cytoplasm. Meanwhile, P-03, P-06, and Lipo2000 took 60 min to achieve a similar signal distribution. These results demonstrate that P-04 peptides are able to gain entry into cells, escape from the endosomal compartment, and translocate plasmid DNA efficiently into the cytoplasm and nuclei within a very short period of time. Furthermore, the efficiency of cellular uptake and endosomal escape for P-04 was improved compared with a previously reported branched cationic polypeptide drug delivery system.^52^

### 3.8. Cytotoxicity

**Fig. 9:**
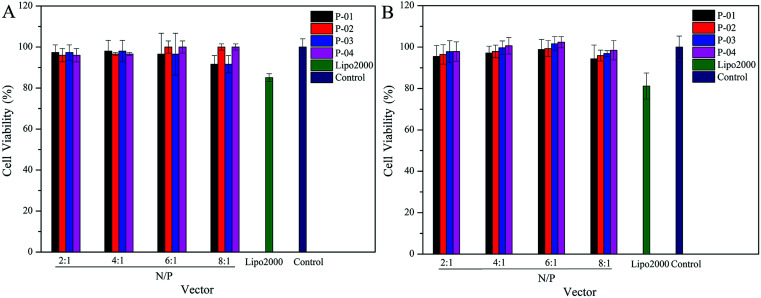
Viability of 293T (A) and NIH-3T3 (B) cells after being treated with different peptide/DNA complexes at N/P ratios ranging from 2 to 8. Data are presented as the mean ± SD (*n* = 5).

Ideally, a gene vector provides good delivery with low cytotoxicity. To assess the cytotoxicity of our peptide/DNA complexes in 293T and NIH-3T3 cells, a CCK-8 kit was used with Lipo2000 and naked plasmid included as controls. At relatively low N/P ratios, no significant cytotoxicity was observed for any of our designed peptide/DNA complexes (Fig. 9). However, when the N/P ratios were increased to greater than 6, the complexes exhibited slight cytotoxicity, potentially due to the increased dosage of the vectors at these higher N/P ratios. However, even at an N/P ratio of 8, the cytotoxicity of the peptide/DNA complexes was lower than that of Lipo2000. Thus, our designed peptide vectors appear to provide gene transfection with low cytotoxicity.

### 3.9. Enzymatic hydrolysis stability

**Fig. 10:**
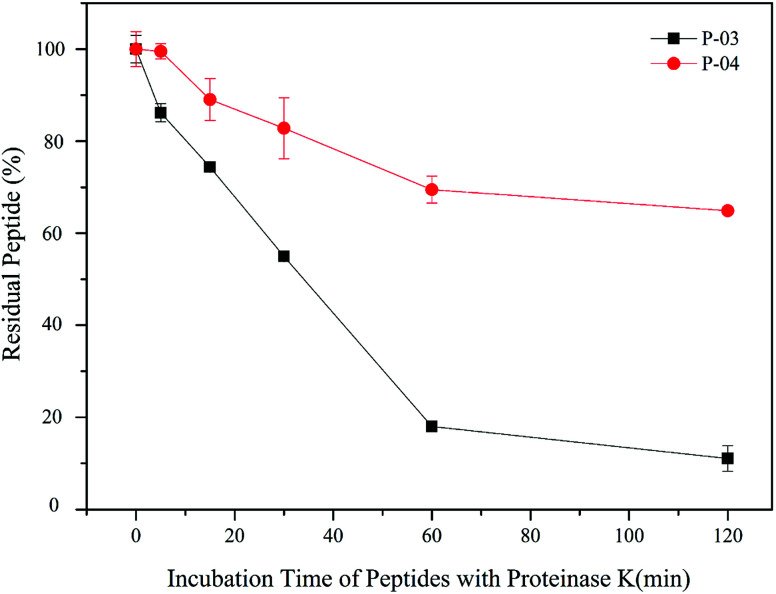
Stability of P-03 and P-04 after being incubated with proteinase K from 0 min to 2 h. Data are presented as the mean ± SD (*n* = 3).

All of our peptide vectors achieved excellent delivery at the cellular level, and this delivery potential is expected to be maintained at an organism level as well. A subset of our peptide vectors also had D-peptides used for their synthesis to increase their resistance to enzymatic hydrolysis. As shown in Fig. 10, 82% of L-peptide P-03 was degraded within 60 min of exposure to proteinase K, while 70% of D-peptide P-04 remained after the same incubation period with proteinase K. Thus, the P-04 D-peptide exhibited greater resistance to enzymatic hydrolysis compared with the P-03 L-peptide.

## Conclusion

Here, we describe the design and synthesis of a series of chimeric multifunctional peptide vectors which contained functional segments, including cell-penetration, histidine-enriched endosomal escape, cysteine and stearyl group segments. By employing a rational arrangement, all of the functional segments exhibited appropriate biological activity. Moreover, in P-04, the segments exhibited synergistic actions to greatly improve the transfection efficiency of this peptide vector, which also exhibited suitable particle size and enhanced uptake *via* cellular internalization mediated by the R_9_ segment. Furthermore, the D-peptides exhibited greater stability than the L-peptides in an enzymatic environment. Thus, the highest transfection efficiency was provided by D-peptide P-04 at an N/P ratio of 6, and this efficiency was approximately 7-fold and 5.5-fold greater than Lipo2000 in 293T and NIH-3T3 cells, respectively. It is anticipated that these findings may advance the use of peptide vectors in the treatment of genetic diseases.

## Conflicts of interest

There are no conflicts to declare.

## Supplementary Material

RA-008-C8RA04101F-s001
