# Supplementary material for: The structure and configuration changes of multifunctional peptide vectors enhance gene delivery efficiency
Source: RSC Adv. 2018 Aug 7;8(50):28356–66. doi: 10.1039/c8ra04101f (PMC9084241; doi:10.1039/c8ra04101f)
Supplement: RA-008-C8RA04101F-s001 [file RA-008-C8RA04101F-s001.pdf]

Electronic Supplementary Material (ESI) for RSC Advances.
This journal is © The Royal Society of Chemistry 2018

Supporting Information

The structure and configuration changes of multifunctional peptide vectors enhance gene delivery efficiency

Sen Yang†, Zhao Meng†, Ziyao Kang, Chao Sun, Taoran Wang, Siliang Feng, Qingbin Meng* and Keliang Liu*

State Key Laboratory of Toxicology and Medical Countermeasures, Beijing Institute of Pharmacology and Toxicology, Beijing, 100850, China.

†These authors contributed equally to this work.

Email: nankaimqb@sina.com, keliangliu55@126.com; Fax: +86-10-68211656; Tel: +86-10-68169363

Table S1 Percentage of α-helical conformation for peptide vectors

Compounds	Percentage of α-helical conformation (%)
P-01	32.18
P-02	34.67
P-03	32.38
P-04	34.60
P-05	24.72
P-06	27.99

Figure S1 RP-HPLC of peptide vectors

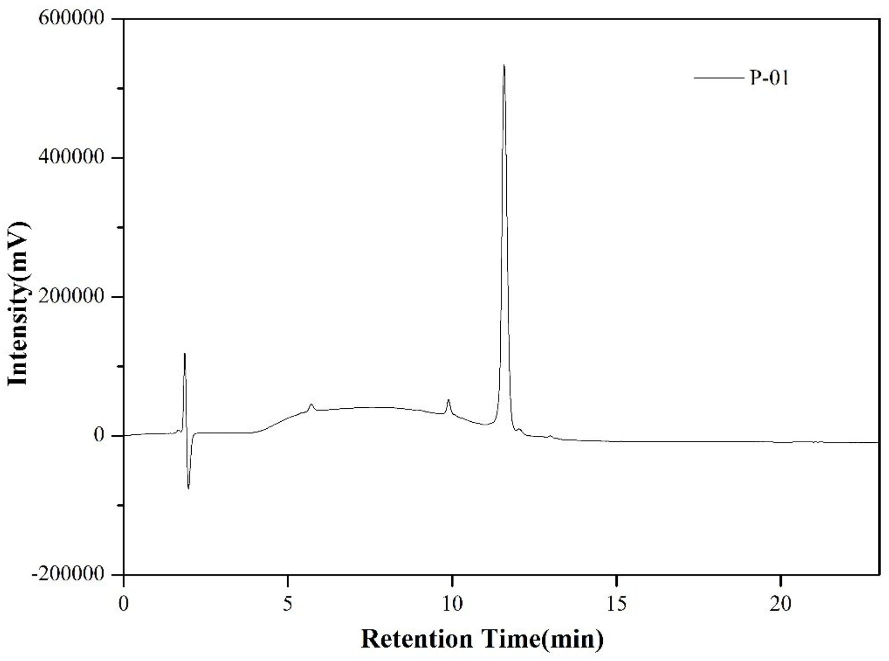

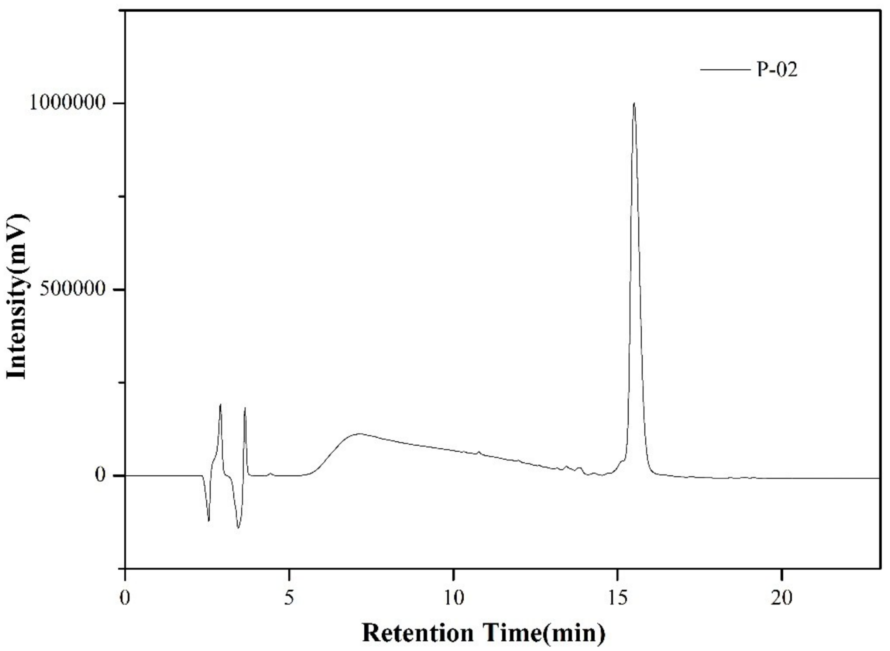
P-02
1000000
500000
0
0
5
10
15
20
Intensity(mV)
Retention Time(min)

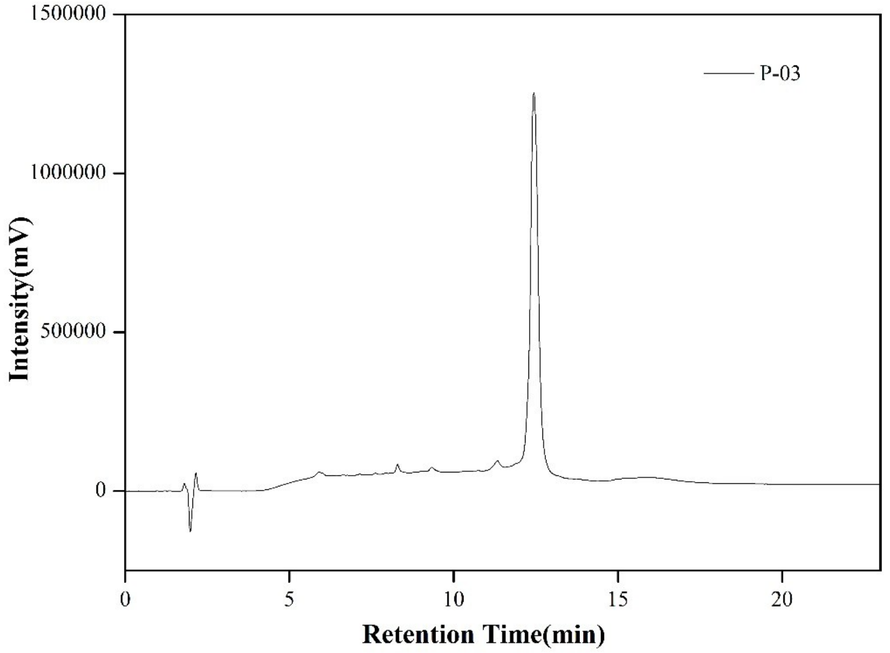
P-03
1500000
1000000
500000
0
0
5
10
15
20
Intensity(mV)
Retention Time(min)

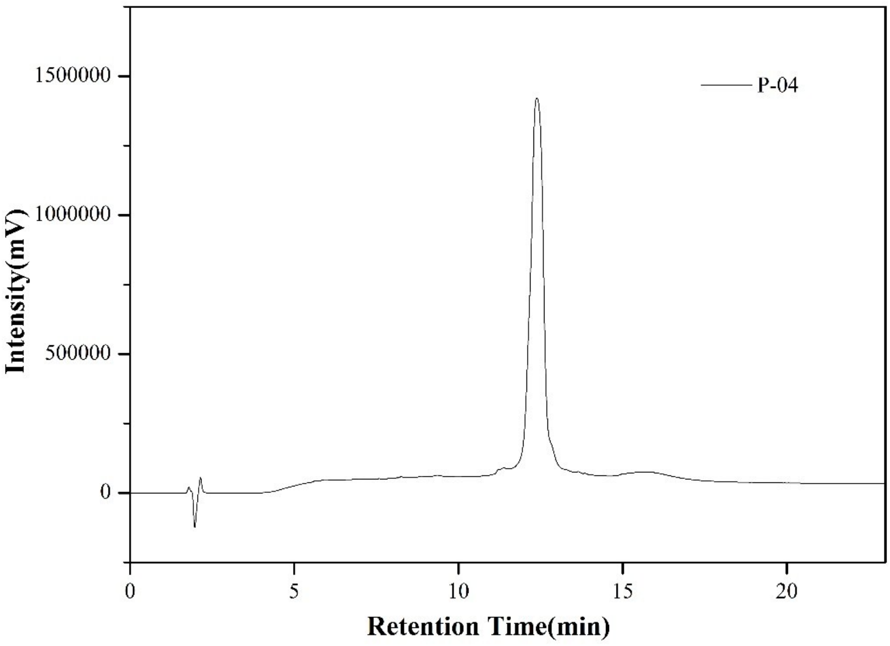
P-04
Intensity(mV)
1500000
1000000
500000
0
0
5
10
15
20
Retention Time(min)

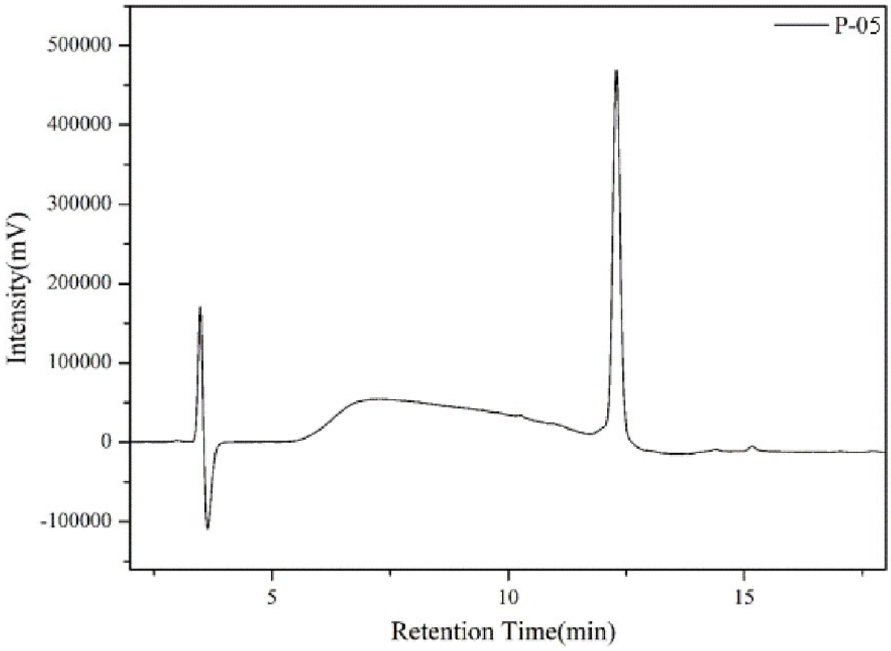
P-05
Intensity(mV)
500000
400000
300000
200000
100000
0
-100000
5
10
15
Retention Time(min)

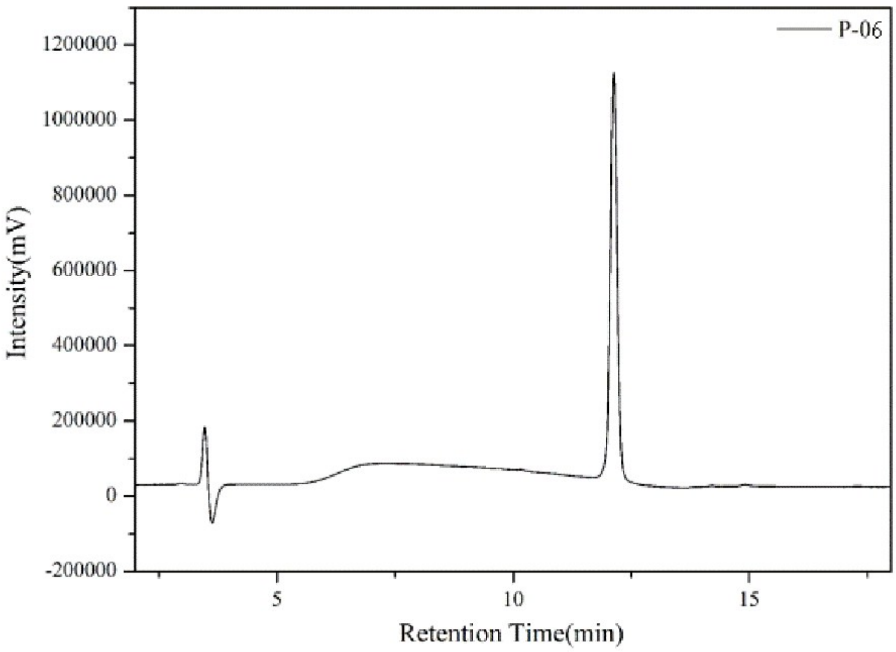
P-06
Intensity(mV)
1200000
1000000
800000
600000
400000
200000
0
-200000
5
10
15
Retention Time(min)

Figure S2 ESI-MS and MALDI-TOF of peptide vectors

P-01

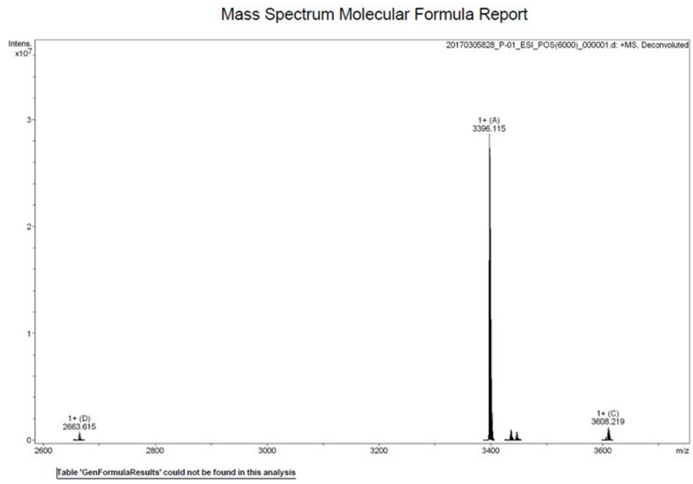

P-02

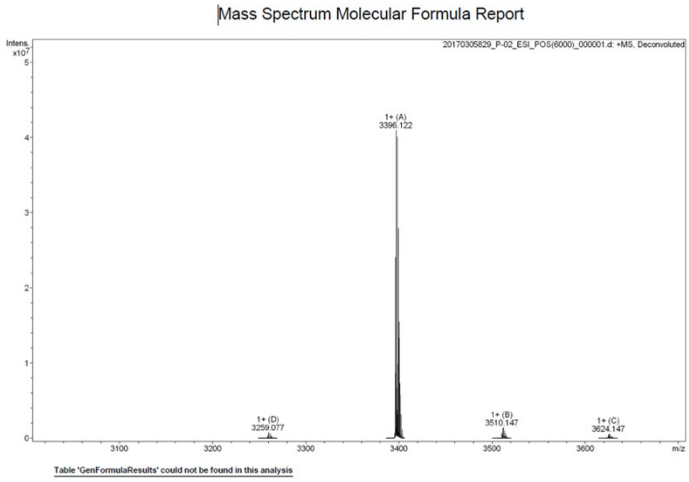

P-03

Mass Spectrum Molecular Formula Report

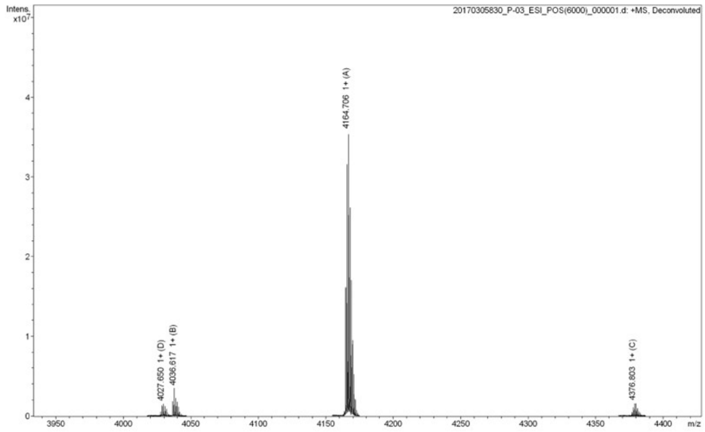

Table 'GenFormulaResults' could not be found in this analysis

P-04

Mass Spectrum Molecular Formula Report

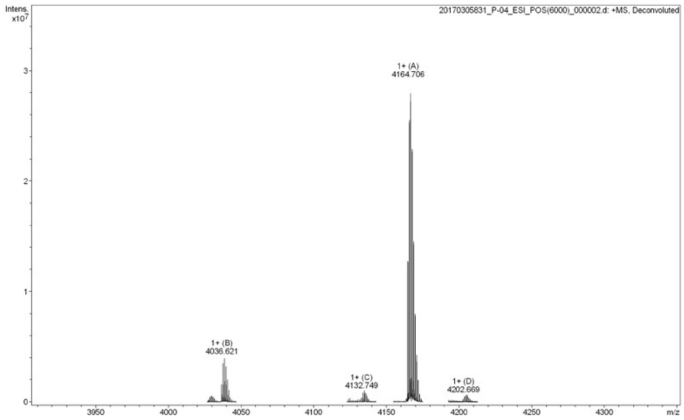

Table 'GenFormulaResults' could not be found in this analysis

P-05

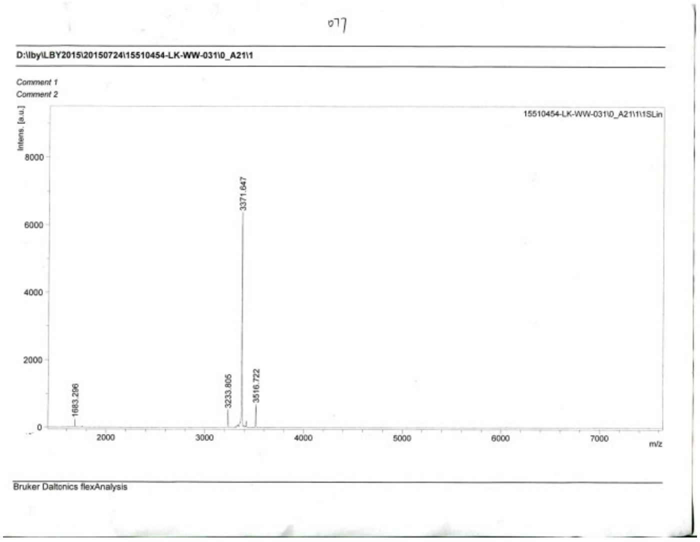
077
D:\lby\LBY2015\20150724\15510454-LK-WW-031\0_A21\1
Comment 1
Comment 2
15510454-LK-WW-031\0_A21\1\1SLin
Intens. [a.u.]
3371.647
1683.296
3233.805
3516.722
m/z
Bruker Daltonics flexAnalysis

P-06

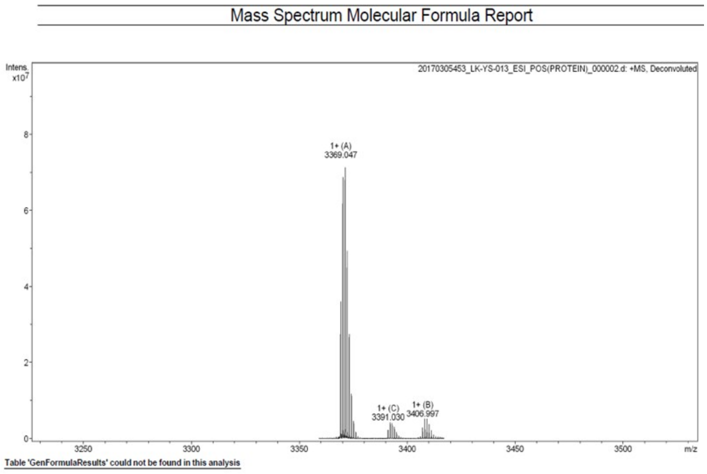
Mass Spectrum Molecular Formula Report
Intens. x10^7
20170305453_LK-YS-013_ESI_POS(PROTEIN)_000002.d: +MS, Deconvoluted
1+ (A)
3369.047
1+ (C)
3391.030
1+ (B)
3406.997
m/z
Table 'GenFormulaResults' could not be found in this analysis
